# Beyond the Heart: A Rare Case of Methicillin-Resistant Staphylococcus aureus (MRSA)-Induced Quadriplegia Without Endocarditis

**DOI:** 10.7759/cureus.92013

**Published:** 2025-09-10

**Authors:** Jawhar Syed, Ethan C Ward, Ashley Sundin, Venkata Vedantam

**Affiliations:** 1 Internal Medicine, East Tennessee State University - Quillen College of Medicine, Johnson City, USA

**Keywords:** acute quadriplegia, cerebral infarcts, epidural abscesses, illicit drugs, mrsa bacteremia, sepsis and septic shock, septic brain embolism, septic emboli, septic pulmonary embolism, vertebral-osteomyelitis

## Abstract

Methicillin-resistant *Staphylococcus aureus* (MRSA) is a major cause of serious community and healthcare-associated infections, with presentations ranging from skin and soft tissue involvement to invasive disease. We report a rare case of MRSA bacteremia in a 56-year-old man with a history of intravenous drug abuse who presented with septic shock and developed acute quadriplegia. Imaging revealed multifocal cerebral septic emboli, extensive vertebral osteomyelitis from C2 to T1, and an epidural abscess causing significant spinal cord compression, all in the absence of identifiable valvular endocarditis. Despite comprehensive multidisciplinary management, the patient's condition progressively deteriorated, and he ultimately succumbed to his illness. This case highlights the potential for MRSA to cause devastating neurological injury through metastatic infection despite no discernible vegetation in the heart. Early recognition, comprehensive imaging, and prompt antimicrobial therapy are essential to identify deep-seated infections and prevent severe outcomes.

## Introduction

*Staphylococcus aureus*, particularly methicillin-resistant *S. aureus* (MRSA), is a major contributor to global mortality and morbidity. This particular bacterial pathogen is a significant source of both community and hospital-based infections. In North America, an estimated 23% of ICU infections are caused by *S. aureus*, with MRSA accounting for 44% of those cases [1]. Among patients with device-associated and surgical-site infections, *S. aureus* is the most common bacterial source for both adult and pediatric patients, 48.4% and 41.9% of these being MRSA, respectively [1]. MRSA colonization can lead to a broad spectrum of clinical presentations, most commonly including skin and soft tissue infections, pneumonia, and in the case of our patient, bacteremia [2]. 

MRSA bloodstream infections are associated with substantial morbidity, mortality, and healthcare burden, with estimated 30-day mortality rates approaching 27% and hospital readmission rates near 22% [3,4]. In this report, we present a rare and devastating case of MRSA bacteremia complicated by vertebral osteomyelitis, epidural abscess, and multifocal cerebral septic emboli, ultimately resulting in acute quadriplegia and death. Notably, this occurred in the absence of identifiable valvular endocarditis, a typical source of septic embolization [5]. This case highlights the importance of maintaining a high index of suspicion for deep-seated and neurologically significant infections in MRSA bacteremia, and underscores the need for early imaging, aggressive antimicrobial therapy, and multidisciplinary management to mitigate life-threatening complications.

## Case presentation

A 56-year-old man with a past medical history significant for recurrent community-acquired MRSA bacteremia secondary to intravenous (IV) drug abuse, most recently complicated by septic pulmonary emboli six months prior, along with type 2 diabetes mellitus, alcohol use disorder, and chronic hepatitis, presented to an outside hospital with acute quadriplegia and septic shock, attributed to MRSA bacteremia confirmed by blood cultures with susceptibility testing (Table 1). He initially complained of several days of generalized fatigue, fever, chills, and elevated glucose levels recorded at home. Shortly after arrival at the outside hospital, he became unresponsive and hypotensive, requiring endotracheal intubation, mechanical ventilation, and sedation. Transesophageal echocardiogram (TTE) was negative for endocarditis. Once hemodynamic stability was achieved, the patient was successfully extubated. However, he demonstrated marked neurological impairment. He was only able to perform eye-opening and brow movements, which prompted a neurologic evaluation. Magnetic resonance imaging (MRI) of the brain and spine was recommended, which required transfer to a tertiary care facility for a higher level of care. The patient was reintubated in preparation for transfer. 

**Table 1:** Susceptibility testing of MRSA-positive blood culture MRSA: methicillin-resistant *Staphylococcus aureus*

Antibiotic	Minimum Inhibitory Concentration (MIC)	Susceptibility
Cefoxitin Screen	Positive ug/mL	Positive
Ceftaroline	0.5 ug/mL	Susceptible
Clindamycin	0.25 ug/mL	Susceptible
Daptomycin	0.5 ug/mL	Susceptible
Erythromycin	≥ 8.0 ug/mL	Resistant
Gentamicin	≤ 0.5 ug/mL	Susceptible
Inducible Clindamycin Resistance	Negative ug/mL	Negative
Linezolid	2.0 ug/mL	Susceptible
Minocycline	≤ 0.5 ug/mL	Susceptible
Oxacillin	≥ 4.0 ug/mL	Resistant
Rifampin	≤ 0.5 ug/mL	Susceptible
Tetracycline	≤ 1.0 ug/mL	Susceptible
Trimethoprim-Sulfamethoxazole	≥ 320 ug/mL	Resistant
Vancomycin	1.0 ug/mL	Susceptible

The patient was transferred to our tertiary center from the outside hospital. On admission, the patient was found to be febrile, and laboratory testing was significant for elevated white blood cells, lactate, and glucose (Table 2). As per the culture and tests done at the outside hospital, an initial diagnosis of septic shock was made, leading to initiation of empiric antibiotics and norepinephrine infusion. 

**Table 2:** Pertinent laboratory values on admission (H) indicates values that are higher than the reference range. (L) indicates values that are lower than the reference range. BUN: blood urea nitrogen

Tests	Patient Values	Reference Ranges
White blood cells (K/uL)	33.7 (H)	3.5-10.5
Hemoglobin (g/dL)	14.5	13.5-17.5
Platelet count (K/uL)	466 (H)	150-450
Neutrophils (K/uL)	30.6 (H)	1.5-7
Lymphocytes (K/uL)	0.5 (L)	0.8-4
Neutrophils (%)	91 (H)	45-75
Lymphocytes (%)	2 (L)	20-50
Lactate (mmol/L)	4.3 (H)	0.5-2
Glucose (mg/dL)	654 (H)	70-99
BUN (mg/dL)	42 (H)	6-20
Creatinine (mg/dL)	1.39 (H)	0.9-1.3

**Figure 1:**
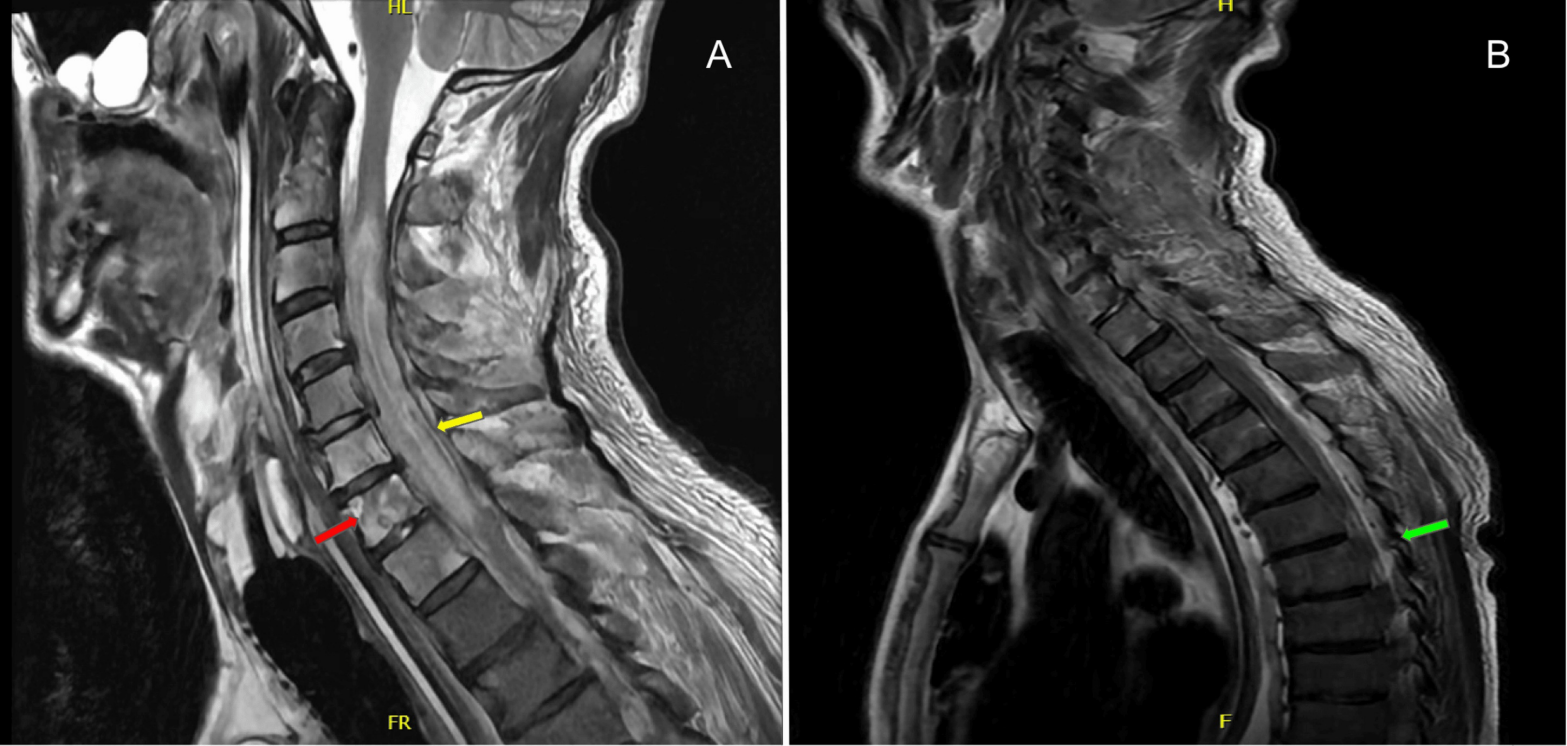
MRI of cervical (A) and thoracic spine (B) illustrating edema and epidural fluid collection suggestive of multilevel vertebral osteomyelitis. Red Arrow: abnormal signal within vertebral bodies; Yellow Arrow: abnormal enhancement along surface of spinal cord; Green Arrow: epidural fluid collection

**Figure 2:**
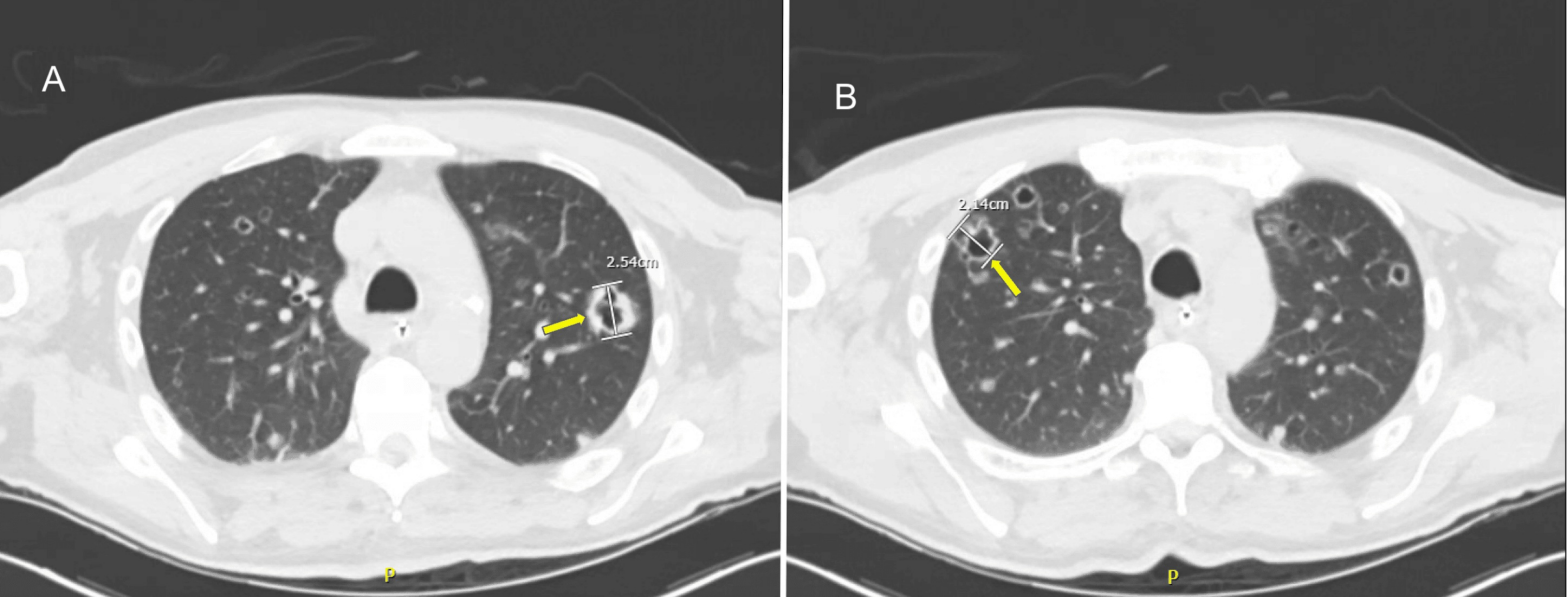
CT scan of chest revealing multifocal cavitary lesions within left (A) and right (B) lobes secondary to septic pulmonary embolization. Yellow Arrows: Cavitary lesions

MRI of the brain was done, which demonstrated multiple foci of punctate acute cerebral ischemia consistent with a central embolic phenomenon. MRI of the spine demonstrated edema from C2 through T1, suggestive of multilevel vertebral osteomyelitis and an epidural fluid collection causing mass effect, most notable at T4-T5, consistent with suspected epidural abscess secondary to bacteremia (Figure 1). Computed tomography (CT) of the chest revealed progression of septic pulmonary emboli compared to previous scans six months prior, as well as multifocal cavitary lesions (Figure 2). TTE did not reveal valvular vegetations or other evidence suggestive of endocarditis, similar to the TTE done at the outside hospital.

The patient was admitted to the intensive care unit with multidisciplinary care, including critical care, infectious disease, neurology, neurosurgery, and palliative care. Neurosurgery deemed the patient was not a good candidate due to the unlikely functional improvement with surgical intervention. The patient underwent aggressive multidisciplinary interventions, including mechanical ventilation, vasopressor therapy, and broad-spectrum antibiotic coverage with vancomycin, ceftriaxone, and ampicillin. Nonetheless, his condition deteriorated secondary to persistent bacteremia, resulting in extensive neurological deficits and multi-organ failure. After discussions with family about the poor prognosis, he was switched to comfort-oriented care. The patient subsequently passed away a few days later. 

## Discussion

MRSA bloodstream infection (bacteremia) is associated with substantial morbidity and mortality. A large meta-analysis and systematic review from 2022 encompassing 341 studies and over 500,000 patients evaluated mortality rates at various time points following the identification of infection; mortality rates were found to be 10.4% at seven days, 18.1% at one month, and 27.0% at three months [3]. Similarly, a 2019 study involving 92,000 patients with bloodstream infections caused by *S. aureus *found 30-day hospital readmission rates to be 22% [4]. Those with MRSA infections experienced significantly increased rates of bacteremia recurrence and prolonged hospital stays. 

The majority of community-acquired MRSA infections (77-90%) present as skin and soft tissue infections, typically in the form of abscesses or cellulitis. Less frequently reported are more invasive infections, such as osteomyelitis, which make up approximately 1% of MRSA infections [2]. MRSA-related osteomyelitis is more commonly seen in the pediatric patient population [2], but is exceedingly rare in the adult population, as in our case. Among such adult cases, vertebral osteomyelitis is one of the most common sites of infection. Clinical course, presentation, complications, and outcomes vary depending on location and the severity of infection. 

MRSA vertebral osteomyelitis in the adult population is associated with high rates of chronic pain, recurrence, mortality, and long-term neurological deficits. One study comparing outcomes in MRSA and methicillin-sensitive *S. aureus* (MSSA) osteomyelitis infections found that MRSA was linked with higher rates of multivertebral body involvement, in-hospital mortality, and surgical debridement rates [6]. An extensive retrospective study analyzing 10,000 cases of MSSA and MRSA osteomyelitis and spondylodiscitis reported that MRSA infections requiring surgery were associated with increased rates of complications, including transfusion requirements, acute kidney injury, urinary tract infections, and post-operative pneumonia [7]. While serious complications such as neurological deficits, spinal instability, deformity, radiculopathy, and paraspinal abscesses stemming from MRSA infection are well documented [8,9], cases of resulting quadriplegia, such as in our patient, are an exceedingly rare finding in the literature. 

Septic emboli in the central nervous system can result in cerebral infarcts, brain abscesses, or meningitis. This phenomenon can be observed in cases of MRSA bacteremia, such as in our patient, and is most commonly associated with bacterial endocarditis, particularly left-sided, which MRSA most commonly causes. Systemic embolization occurs in 22-50% of patients with endocarditis, with up to 65% of embolic events affecting the central nervous system, most commonly in the distribution of the left middle cerebral artery [10]. In our patient’s case, multiple transesophageal and transthoracic echocardiograms performed during workup for endocarditis were found to be negative, despite persistent MRSA bacteremia. The presence of numerous brain infarctions seen on MRI from MRSA septic embolization in the absence of confirmed cardiac involvement is rare and not well quantified in the literature. 

Septic embolization in the setting of MRSA bacteremia without endocarditis most commonly arises from deep tissue infections such as osteomyelitis, pyomyositis, or other soft tissue abscesses [11]. Other common sources that may go undetected, likely in the case of our patient, include septic thrombophlebitis in the context of indwelling catheters, vascular access points, or directly from injected drug use [12]. Detection of these sources may be challenging, especially in the setting of absent clinical signs. The risk of metastatic septic embolization has been found to be higher in community-acquired cases and in the presence of foreign bodies or persistent bacteremia [13]. 

Treatment of MRSA bacteremia, particularly in complex cases such as this one, centers on managing underlying sepsis with appropriate antimicrobial therapy and identification of septic embolic metastatic complications. Early diagnosis, combined with a comprehensive workup, is crucial for achieving improved patient outcomes. This includes obtaining prompt blood cultures, measuring inflammatory markers, and performing serial cultures to assess for persistent infections [14]. According to the Infectious Disease Society of America (IDSA) and recent clinical trials, vancomycin with target trough concentrations of 15 mg/L or greater is recommended in the setting of deep-seated infections such as vertebral osteomyelitis. Daptomycin is considered a suitable alternative in patients with vancomycin intolerance or nephrotoxicity [15]. 

MRI of the brain is the most sensitive imaging modality for the detection of cerebral infarcts secondary to septic emboli, particularly when head CT is unrevealing [16]. MRI of the spine is also considered the gold standard imaging modality for detecting vertebral osteomyelitis in adults with MRSA bacteremia, with or without cardiac involvement [17]. In cases of paraspinal or epidural abscesses secondary to MRSA vertebral osteomyelitis, drainage of the abscess was associated with higher cure rates. Surgical intervention is indicated in patients with neurologic compromise, spinal instability, abscesses not amenable to percutaneous drainage, or failure of medical therapy [18]. 

Patient outcomes are closely correlated with timelines and the adequacy of therapy. Delayed or suboptimal treatment is associated with an increase in mortality, higher risks of relapsing or persistent infection, and diminished functional recovery. In studies of MRSA vertebral osteomyelitis, cure rates exceeded 80% with at least eight weeks of effective therapy combined with adequate source control. In contrast, rapid relapse rates were observed in patients receiving shorter courses or those with undrained abscesses [18]. The Society of Critical Care Medicine highlights that delays in initiation of effective MRSA therapy beyond 24-48 hours are associated with increased mortality [14]. 

## Conclusions

This case illustrates the rare but severe complication of quadriplegia resulting from septic involvement of brain and vertebral osteomyelitis secondary to MRSA bacteremia, occurring in the absence of valvular vegetations. The likely mechanism involves bacteremia-associated septic thrombi originating from an undetermined extracardiac focus. Even in the absence of endocarditis, thorough evaluation for other potential sources or metastatic spread of infection is critical in cases of MRSA bacteremia, as it directly informs appropriate medical and surgical management. This case underscores the critical importance of early antimicrobial therapy and thorough investigation for metastatic septic emboli, including evaluation of the endocardium, vertebral column, and central nervous system. Comprehensive assessment of these potential infection sites is essential in the management of MRSA bacteremia to minimize long-term neurological deficits, persistent infections, mortality, and rehospitalization.
